# Nutraceutical, Anti-Inflammatory, and Immune Modulatory Effects of *β*-Glucan Isolated from* Yeast*

**DOI:** 10.1155/2017/8972678

**Published:** 2017-08-23

**Authors:** Umar Bacha, Muhammad Nasir, Sanaullah Iqbal, Aftab Ahmad Anjum

**Affiliations:** ^1^Department of Food Science and Human Nutrition, University of Veterinary & Animal Sciences, Lahore, Pakistan; ^2^Department of Microbiology, University of Veterinary & Animal Sciences, Lahore, Pakistan

## Abstract

*β*-Glucan is a dietary fibre, found in many natural sources, and controls chronic metabolic diseases effectively. However, *β*-glucan from the* yeast* has rarely been investigated. Objectively, conditions were optimized to isolate *β*-glucan from the yeast (max. 66% yield); those optimized conditions included 1.0 M NaOH, pH 7.0, and 90°C. The purity and identity of the isolated *β*-glucan were characterized through FT-IR, SEM, DSC, and physicofunctional properties. The obtained results from DSC revealed highly stable *β*-glucan (m.p., 125°C) with antioxidant activity (TAC value 0.240 ± 0.0021 *µ*g/mg, H_2_O_2_ scavenging 38%), which has promising bile acid binding 40.463% and glucose control (in vitro). In line with these results, we evaluated the in vivo anti-inflammatory potential, that is, myeloperoxidase activity and reduction in MDA and NO; protective effect on proteins and keeping viscosity within normal range exhibited improvement. Also, the in vivo cholesterol binding and reduction in the skin thickness by *β*-glucan were highly encouraging. Finally, our results confirmed that yeast *β*-glucan is effective against some of the inflammatory and oxidative stress markers studied in this investigation. In general, the effect of 4%  *β*-glucan was more noticeable versus 2%  *β*-glucan. Therefore, our results support the utilization of *β*-glucan as a novel, economically cheap, and functional food ingredient.

## 1. Introduction

The prevalence of chronic diseases has escalated rapidly in recent years worldwide. There are persisting long term noncommunicable conditions, challenging the public health, increasing morbidity, and affecting human productivity. They are the most common, costly to cure, and major cause of unnecessary death. Cardiovascular diseases, diabetes, cancer, oxidative stress [1, 2], and many more have significantly contributed to the burden on public health because of the disability and death they cause. Chronic diseases respond well to dietary interventions and thus they are known as lifestyle related diseases. So far, dietary fibre with linkages 1–3 and 1–4 (glycosidic bonds) has demonstrated positive role in managing chronic disease. *β*-Glucan shares features of dietary fibres, that is, 1–3 and 1–6 (glycosidic bonds): therefore, it can be used in the management of chronic diseases. There have been plenty of studies published [3–5] that reported the effectiveness of *β*-glucan in diabetes and cardiovascular diseases.

Like other polysaccharides and dietary fibres, *β*-glucan mechanism of action includes reduction in nutrient absorption and improving viscosity of intestinal contents. Further, *β*-glucan can be a potential source for the fermentation by the microbes in the small intestine [6] and may produce prebiotic effect. Moreover, their ability in enhancing host immune response is well documented. *β*-Glucan of different sources differs based on their structure and molecular size and due to these differences, the functionalities (viscosity, fermentability, solubility, etc.) of *β*-glucan vary, leading to different physiological outcomes. Unlike barley and oat,* yeastβ*-glucan is the least investigated substance and hence the present paper has put an effort to fill the gaps in the literature through investigating immune compromised animals (rats) following dinitrochlorobenzene (DNCB) exposure in those rats.

Regarding their isolation, it can be done from barley [7, 8] oat, fungi, yeast, and some bacteria [2]. Although yeast contains appreciable quantity of *β*-glucan the isolation is difficult as the* yeast* cell wall is compact and rigid. To break this dimension, coprocess consisting of hot-cold water extraction with or without acid extraction can be used [2], but the process is time consuming, resulting in product with low purity and high chemical cost. So, the second prime objective of the current investigation was to design environment friendly process that could produce more cost-effective *β*-glucan from cheap and easily available* yeast*.

## 2. Materials and Methods

Commercial *β*-glucan and* yeast* were purchased from the local supermarket and stored under room temperature till further analyses.

### 2.1. Yeast Biomass Production

The yeast biomass was produced according to our previous method [9] with little modification. Briefly, from the stock culture (2%) of* Saccharomyces cerevisiae*, 1.0 mL of the culture was transferred to Erlenmeyer flasks. Prior to inoculation, broth was added to the culture vessel. The composition is given/L: 4.0 g beef extract, 8.0 g peptone, 5 g sodium chloride, 16 g glucose, and 200 g potato extract. This was followed by inoculation using an incubator at 30°C for 5 days. Moreover, biomass produced was calculated by filtering the samples through filter paper (0.2 *μ*m) and washing two times with distilled water followed by washing in 0.9% NaCl solution. Finally, the biomass was dried in oven (105°C).

### 2.2. Isolation and Extraction of *β*-Glucan

*Yeast* biomass on dry weight basis, that is, three grams (3.0 g), was placed in a flask, and then 30 mL of sodium hydroxide (NaOH) solution having different molarity was added. The effect of 1, 2, 3, and 4 molar NaOH solutions was tested on *β*-glucan extraction. The solution containing* yeast *biomass was heated in a water bath for fixed 3 hrs. The second experiment was conducted to understand the effect of temperature on *β*-glucan extraction. The effect of 90, 70, 50, and 35°C, respectively, temperatures was tested. The third part of the *β*-glucan isolation from the* yeast* involved the effect of pH; for this purpose, 4, 7, 9, and 12 pHs were tested for the *β*-glucan extraction. According to the treatment plan, after a brief heating for 3 hrs, the slurry was cooled to ambient temperature and then centrifuged (15,000*g*, 30 min). The supernatant was collected in a flask and then ethanol (90% v/v) was added in equal quantity. The flasks were placed in refrigerator overnight (4°C). This step resulted in *β*-glucan precipitation [8]. The precipitated *β*-glucan was freeze-dried and weight was calculated (%).

### 2.3. Characterization of *β*-Glucan

The in vivo and in vitro characterization of* yeastβ*-glucan was carried out through the following experimentation.

#### 2.3.1. Fourier Transforms Infrared Spectroscopy (FT-IR) of *β*-Glucan

The FIR analysis of the samples was carried through FT-IR-8300 instrument (Shimadzu), according to the method [11] with little modification. The dried samples were mixed with KBr powder and transparent blend in the form a film/tablet was made by applying 10 tons of pressure using hydraulic piston. FT-IR spectra were recorded at a resolution of 2 cm^−1^ between 4000 cm^−1^ and 400 cm^−1^. A total of 32 scans were performed.

#### 2.3.2. Staining and Scanning Electron Microscopy (SEM)

The procedure is carried out in accordance with [12]. The alkali treated* yeast* cells and those not treated were subjected to staining to differentiate between lysed and unlysed cells. For the SEM analyses, samples were kept on stubs using carbon tape; images were generated with SEM (Scanning Electron Microscope) at an acceleration voltage of 10 kV.

#### 2.3.3. Differential Scanning Calorimetry (DSC) Analyses

*β*-Glucan and related samples were analyzed through instrument Differential Scanning Calorimeter (TA-Q20 Inc, US), according to the method [13], with little modification. Sample (8.0 mg) was put into an aluminum pan and sealed hermetically. The temperature information is as follows: equilibrium temperature at 10°C for 1.0 min and the temperature was raised to 150°C @ 3°C/min and at the mentioned temperature samples were held (10.0 min). In this way melting/separation were calculated by the TA software.

#### 2.3.4. Chemical Analysis

Extracted *β*-glucan and commercial *β*-glucan in triplicate were freeze-dried followed by grinding and passing through a 1 mm mesh screen. The samples were then analyzed for dry matter (DM), crude protein (CP), ether extract (EE), crude fibre (CF), ash, and nitrogen-free extract, NFE [14].

#### 2.3.5. Bile Acid Binding Activity of *β*-Glucan (In Vitro)

This experimentation was performed according to the procedure [15]. According to the composition of human bile acids, the following mixture (36 mmol/L) was prepared in phosphate buffer (0.1 M): taurodeoxycholic acid, glycocholic acid, glycodeoxycholic acid, glycochenocholic acid, and taurocholic acid. The pH of the mixture was kept as 6.3. For the working solution, the stock solution was diluted (0.72 *µ*mol/mL) using the dilution formula.

#### 2.3.6. Antioxidant Property of *β*-Glucan

Total antioxidant capacity (TAC) was measured. The mechanistic approach was to reduce Mo(VI) to Mo(V) in the presence of a reducing agent, which is in this case *β*-glucan. For this purpose, samples (0.5 g) were mixed in tubes charged with 15 mL DMSO, boiled in water bath for 4 hours with stirring from time to time. The slurry was cooled and centrifuged (3000 rpm, 15 min). The supernatant was mixed with 400 *µ*L of reagent solution, 170 *µ*L distilled water, and appropriate quantity of buffer so that final volume adjusted to 1000 *µ*L. The tubes were incubated for 90 min (95°C) using a water bath followed by cooling to room temperature. The samples tubes were analyzed through spectrophotometer at 695 nm against blank solution in triplicate [16].

#### 2.3.7. Scavenging (H_2_O_2_) Activity of *β*-Glucan

Hydrogen peroxide was prepared in phosphate buffer (50 mM, pH 7) according to [17]. Its molar concentration was determined (240 nm) using H_2_O_2_ molar extinction coefficient. Ascorbic acid or sample (0.1 mL) of different strength was then added to test tubes. Volumes in these tubes were made as 0.4 mL by the addition of phosphate buffer. This was followed by adding 0.6 mL H2O2 solution. Following ten-minute incubation and vortex of the tubes their absorbance was carried out at 240 nm against a blank phosphate buffer having no H_2_O_2_ and the % scavenging was calculated = (1 − *As*/*Ab*) × 100: absorbance without sample = *Ab*; absorbance with sample = *As*.

#### 2.3.8. Solubility and Swelling Percentage

The *β*-glucan solubility and swelling percentage was determined according to the procedure [18]. *β*-Glucan (0.2 g) was mixed with 10 mL water in tube and incubated at 95°C. The suspension was stirred continually for 30 min for uniform temperature followed by rapid cooling to 20°C. The tubes were then centrifuged (2200 ×g, 15 min); this results in separating supernatant from the pellets. This was followed by taking the supernatant in a crucible and drying at 100°C in oven for 4 h for solubility while the wet pellet was weighted followed by drying in oven for swelling property.

#### 2.3.9. Functional Properties

Functional properties such as water and oil absorption capacities, least gelation concentration [19], and bulk density [20] of commercial and extracted *β*-glucan were determined according to their respective procedures.

#### 2.3.10. Glycaemic Response Evaluation (In Vitro) Study

The *β*-glucan and yeast cell wall were analyzed for glycaemic response during in vitro digestion method. The experiment was repeated at least three times. The procedures of this experiment are based on the previously reported method [21] that mimics the human digestive track.

### 2.4. Biological Characterization

#### 2.4.1. Preparation of 1-Chloro-2,4-dinitrobenzene Preparation (DNCB)

Freshly prepared DNCB (1%) was dissolved in acetone. For the sensitization of rats, exactly 50 *µ*L DNCB was (applied to 12 mm size paper disc) placed on rats left side, shaved abdominal skin. Control normal rats were similarly treated with acetone only. One week after the sensitization, the same concentration of DNCB (challenging dose) was applied on the right side of the abdomen to the shaved area. The skin diameter on the right side through Day 1 to Day 5 was measured in four groups of rats [22]. The information regarding experimental rats is as follows:

  Group 1 = rats fed on control normal diet
  Group 2 = rats fed on control normal diet and treated with DNCB
  Group 3 = rats fed on 2% *β*-glucan diet and treated with DNCB
  Group 4 = rats fed on 4% *β*-glucan diet and treated with DNCB

#### 2.4.2. Animals Study

All experiments related to animal trials conducted in this study are approved by the Institutional Review Board of the University on Animals Care. Four groups of male Sprague Daley rats (*n* = 5 per group), eight weeks old, were used in this study. The rats were kept at 23 ± 1°C with a 12 hrs light/dark cycle and had free access to water and diet. The rats were given a normal diet for one week before the experiments in order to stabilize them. The dietary protocol (Table 1) was carried out for 14 days and the skin thickness was measured after challenging dose of DNCB. At the end of the experiment, overnight fasting rats were harvested and blood and organs were separated.

#### 2.4.3. Blood Analysis

The overnight fasting rats were anesthetized and the whole blood samples (3.0 mL) were obtained directly from the heart and the blood was kept in vacutainer tubes. The blood was analyzed for blood chemistry using a hematology analyzer following the manufacturer's instruction.

#### 2.4.4. Myeloperoxidase and NO Estimation

Determination of myeloperoxidase (MPO) activity in skin tissues was carried out using a spectrophotometer and the MPO activity was carried out [23] while NO was quantified enzymatically, through nitrate reductase that reduces nitrate to nitrite, followed by colorimetric estimation of nitrite at 550 nm using a spectrophotometer.

#### 2.4.5. Statistical Analysis

The data related to skin thickness was analyzed through repeated measure (ANOVA) using GraphPad Prism 5 (GraphPad Software, Inc.7825, USA) and the data related to the isolation, blood parameters, inflammation, and so forth was analyzed through one-way analysis of variance. Comparison between two different samples was analyzed through* t-*test wherever it was necessary. Each experiment was repeated 3 times, at least. The results obtained from the tests were expressed as a mean ± standard deviation (SD) with values of *P* < 0.05 considered significant.

## 3. Results and Discussion

### 3.1. Process Optimization and Isolation of *β*-Glucan

#### 3.1.1. Yeast Biomass Was Used to Isolate *β*-Glucan

**Figure 1:**
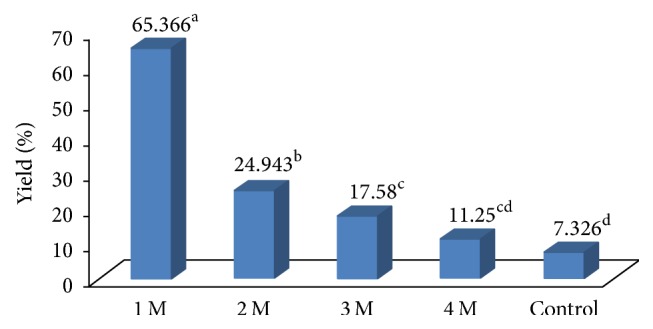
Effect of NaOH (M) concentration on *β*-glucan yield. Control treatment involved extraction of *β*-glucan under hot water extraction; temperature was kept as 90°C throughout this experiment; incubation time was 3 hrs. One-factor analysis was used to compare the mean at *α* = 0.005 using GraphPad Prism version 5; *n* = 3. Different superscript letters, resulting from post hoc test after application of statistical model, show that treatments are statistically different from each other.

The extraction procedure is reported in the method section of this paper. The effect of sodium hydroxide concentration on the extractability of *β*-glucan is presented (Figure 1). The results demonstrated a significant effect of NaOH concentration (*P* ≤ 0.0001) on *β*-glucan yield. As evident from the results presented, NaOH at lower concentration (1.0 M) was effective in releasing *β*-glucan from the* yeast* biomass versus NaOH concentration used at a higher level and control treatment. The control treatment involved water extraction. The extraction process was repeated at least six times, at 90°C for 3 hrs.

**Figure 2:**
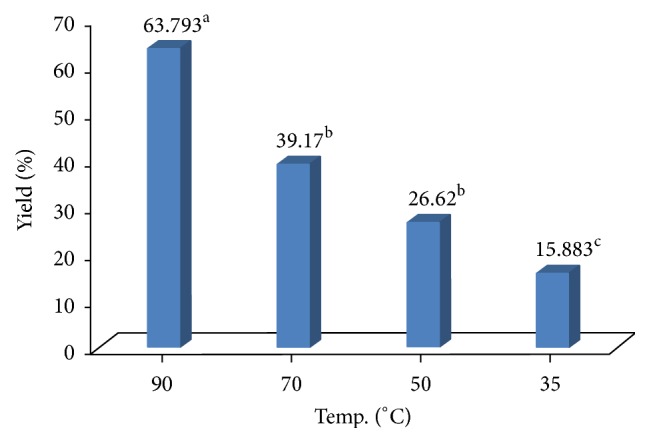
Effect of temperature on *β*-glucan yield (%). Extraction of *β*-glucan at different temperature. Incubation time was 3 hrs. One-factor analysis was used to compare the mean at *α* = 0.005 using GraphPad Prism version 5; *n* = 3. Different superscript letters, resulting from post hoc test after application of statistical model, show that treatments are statistically different from each other.

**Figure 3:**
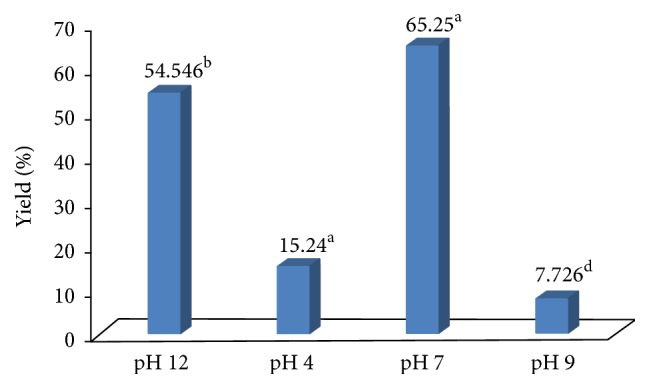
Effect of pH on *β*-glucan yield (%). Extraction of *β*-glucan at different pH. Incubation time was 3 hrs. One-factor analysis was used to compare the mean at *α* = 0.005 using GraphPad Prism version 5; *n* = 3. Different superscript letters, resulting from post hoc test after application of statistical model, show that treatments are statistically different from each other.

The results given (Figures 2 and 3) clearly show significant effects (*P* ≤ 0.0001) of temperature and pH on *β*-glucan yield. It is observed that 90°C and pH 7.0 are the suitable range at which maximum *β*-glucan can be extracted using 1.0 M NaOH solution. Our results are well agreed with recent findings [24] where they have reported 64.56 ± 1.25% *β*-glucan yield using hot NaOH (0.5 M) but they have activated the extraction process for 24 h (yeast autolysis) before actual hot NaOH extraction. Similarly, researcher has isolated *β*-glucan from* Actinomyces rutgersensis 88 yeast* [12] with similar efficiency but the extraction time was high (4hrs). Therefore, current study has reduced the extraction time and simplified the extraction procedures which are cost-effective and environment friendly.

### 3.2. Characterization of *β*-Glucan

#### 3.2.1. Fourier Transforms Infrared Spectroscopy (FT-IR)

**Figure 4:**
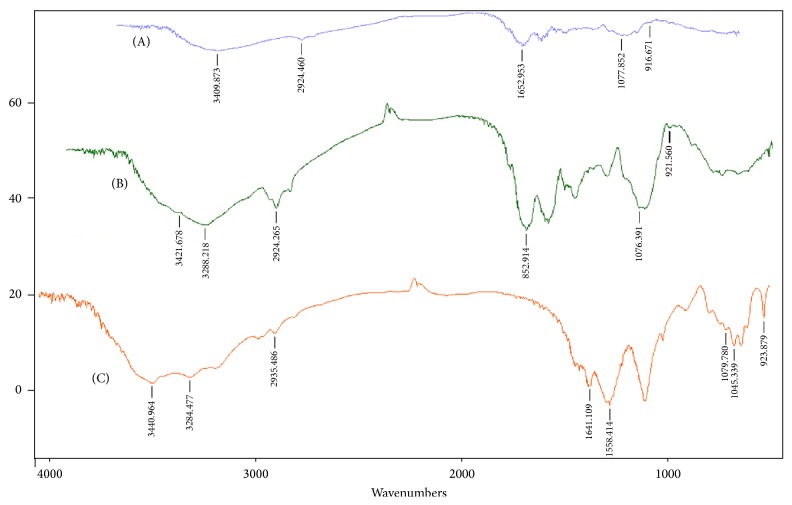
FT-IR analyses: (A) yeast cells, (B) commercial *β*-glucan, and (C) extracted *β*-glucan.

Figure 4 describes the FT-IR analyses of yeast cell (containing proteins and other related structures) and commercial and purified-isolated *β*-glucan. The yeast cells presented five bands (Figure 4(A)); a wide band at 3409.873 cm^−1^ belongs to O-H stretching, as the outer cell wall of* yeast* is predominantly composed of carbohydrates. The polysaccharides are rich in OH groups. The -OH stretching can be found around 4000–3000 cm^−1^ [8] which means the peak 3409.873 cm^−1^ in our case is that of O-H groups. The second wide spectral fingerprint can be seen at 2924.460 cm^−1^, indicating CH and CH_2_ stretching. These findings are in line with [8] that reported bands around 3000–2840 cm^−1^ for CH and CH_2_ stretching.

The 3rd peak at position 1652.953 cm^−1^ could be due to NH groups, indicating the presence of the proteins [10]. For the last two bands, one at 1077.852 cm^−1^ is due to COC bond, that is, glycosidic bonds with cyclic structure [8], and 916.671 cm^−1^ band is probably indicating repeated glucose units like the way found in starch. These observations are indicated previously [24]. In general, carbohydrate composed structure can be identified through bands around 1040 cm^−1^ representing CO bond, 2940 cm^−1^ (CH bond), peak around 3400 cm^−1^ is due to OH stretch [25] and bands (987 cm^−1^, 899 cm^−1^) related to *β*-glycosidic anomeric groups, which in this study was found around 916.671 cm^−1^ in* yeast* cells. These observations are also in line with the recent study [2] and thus confirm the presence of* yeast*.

 Figures 4(B) and 4(C) illustrated the detailed spectral information related to commercial and isolated *β*-glucan from the yeast cells, respectively, in this experiment. As evident from Table 2 commercial (3421.678 cm^−1^) and isolated *β*-glucan (3440.964 cm^−1^) have wider bands than the bands observed in* yeast* cell (3409.873 cm^−1^). This might indicate that -OH groups were embedded in the* yeast *cell wall and thus the bands were less wide. This might be due to the interaction of hydroxyl groups with proteins present in the* yeast* cell wall. Conversely, commercial and isolated *β*-glucan have shown limited proteins; thereby the -OH group was exposed to the environment due to the cell wall disruption thus presenting better stretching of this group.

Moreover, the peak intensity of the -OH group is higher and wider in the case of isolated *β*-glucan relative to the commercial *β*-glucan. Another advantage associated with isolated *β*-glucan was the stretching vibration of -CH_2_ groups, which exhibited a wide band versus the stretching vibration of -CH_2_ groups in the* yeast* cell and commercial *β*-glucan. This implies the better extraction procedures carried out in this experiment. Furthermore, the protein (-NH) bands in the isolated *β*-glucan (1641.109 cm^−1^; 1558.14 cm^−1^) were shifted towards lower wavenumbers than the* yeast* or commercial *β*-glucan (Table 2). This indicates that the isolated samples have less protein and hence confirms the purity of the isolated *β*-glucan. More importantly, the spectral absorption exhibited by the isolated *β*-glucan at position number 1079.780 cm^−1^, 1045.339 cm^−1^, and 923.879 cm^−1^ confirms the presence of glycosidic bonds, demonstrating that *β*-glucan is not broken.

#### 3.2.2. Scanning Electron Microscopy and Gram Staining

**Figure 5:**
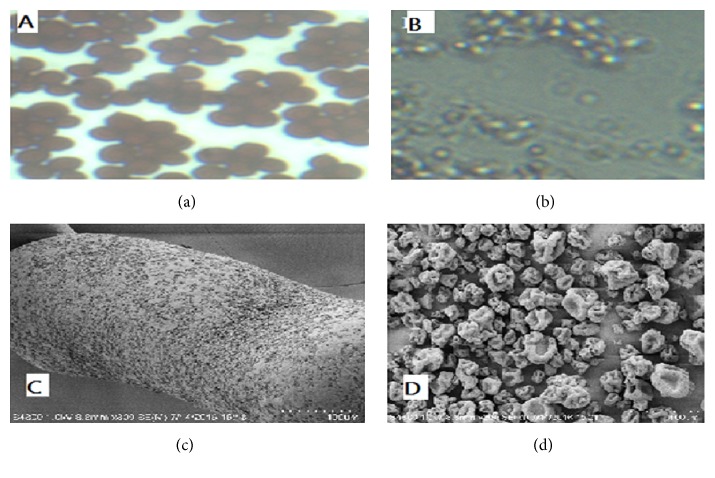
Gram staining and SEM micrograph of yeast cell and extracted *β*-glucan; (a)* yeast* cell, (b) yeast cells lysed with 1 M NaOH at 90°C for 3 hrs, (c)* y*east cells SEM photograph, and (d) extracted *β*-glucan.

Gram staining of intact yeast cells (Figure 5(a)) and broken yeast cells (Figure 5(b)) is presented. The NaOH treated yeast cells are completely broken and their lyses can be seen (Figure 5(b)). These figures confirmed that alkaline treatment has disrupted* yeast* cell wall, as evidenced by smaller and visible fragment (Figure 5(b)) versus the untreated (Figure 5(a)) yeast cells. These findings are in line with the earlier study [12].  Figure 5(c) presents SEM micrograph of yeast cell while Figure 5(d) shows the SEM micrograph of extracted *β*-glucan. The extracted *β*-glucan had a spongy and porous structure. Moreover, there is no indication of the presence of cell wall traces (Figure 5(d)) and that the extracted *β*-glucan presented uniform structures; most of these were larger and in uniform distribution. The present extraction method offers more protection to *β*-glucan structure, which seems better than the methods reported by [8, 25].

#### 3.2.3. Differential Scanning Calorimetry (DSC) Analyses

**Figure 6:**
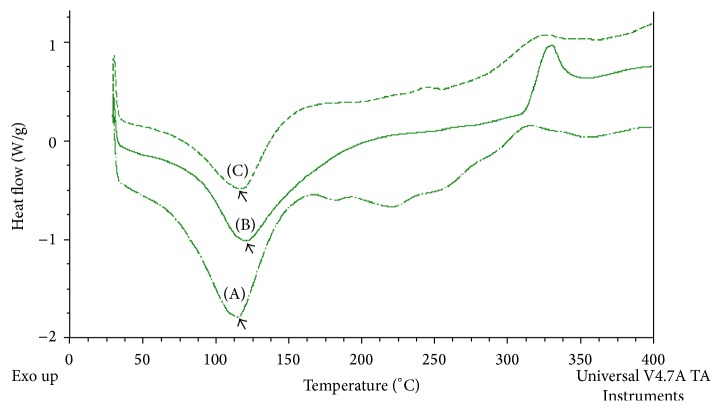
DSC analyses: (A) yeast cell, (B) extracted *β*-glucan, and (C) commercial *β*-glucan.

Differential scanning calorimetry (DSC) techniques were used to characterize samples for their stability over wide temperature range (*β*-glucan,* yeast* cell) as can be viewed from Figure 6. Melting peak around 116°C in the DSC curve (Figure 6(A)) suggested the weight loss and decomposition in* yeast* cells at that temperature while extracted *β*-glucan exhibited melting peak around 125°C, indicating the better thermal stability versus* yeast* cells (Figure 6(B)). Furthermore, the thermal stability of commercial *β*-glucan was found to be little lower (around 122°C) than extracted *β*-glucan (Figure 6(C)). Relative to the* yeast* cells decomposition, *β*-glucan had more compact order forms of the molecules which might be the underlying mechanism for their enhanced thermal stability.

Moreover, an exothermic broad peak appears at 340°C, indicating extracted *β*-glucan oxidation (Figure 6(B)). This implies that *β*-glucan molecules start thermal melting around 125°C though, but the molecule withstands the heat till the temperature reaches 340°C. In general, thermal analysis clearly indicated strong thermal behavior of the *β*-glucan investigated in this study.* Yeast* cells and both types of the *β*-glucan initiated their decay at temperatures below 125°C, but *β*-glucan offers protection in case where high temperature application is warranted. However, a temperature beyond 340°C could be detrimental and will likely offer no resistance as at that temperature their oxidations occur. Therefore, the obtained results indicated that both *β*-glucan types can be used in numerous industrial applications, particularly, as an antioxidant, viscosity modifiers, and water and oil binding agents.

#### 3.2.4. Composition (%; DW) and Properties of Commercial and Extracted *β*-Glucan

Proximate chemical composition of commercial and isolated *β*-glucan is depicted in the Table 3, indicating no statistically significant difference (*P* ≥ 0.05). Fluctuated results are reported regarding the protein contents in the yeast cell wall preparations [26]. Reduced proteins in the *β*-glucan reflect the intensity of yeast cell disruption [27]. The extracted *β*-glucan has presented a decreased protein level in our work indicating effective disruption of the cell wall which is also confirmed through the FT-IR analyses of the *β*-glucans.

Loose and packed bulk density (2.17 ± 0.3, 1.42 ± 0.4), respectively, of commercial *β*-glucan were significantly higher than loose and packed bulk densities 0.698 ± 0.0 and 0.379 ± 0.0, respectively, of extracted *β*-glucan (*P* ≤ 0.05; Table 3). The slightly higher water absorption capacity of commercial *β*-glucan relative to extracted *β*-glucan is due to the higher protein contents in the commercial *β*-glucan. The water absorption potential in both cases is comparable to the WAC of barely *β*-glucan and sugar beet fibre 3.28 g/g 4.56 g/g, respectively [28], and therefore extracted *β*-glucan can be used to avoid syneresis in many foods.

Regarding the *β*-glucan water solubility (%), extracted *β*-glucan presented 7.9 ± 6.0% water solubility versus commercial *β*-glucan, 8.6 ± 8.7% (Table 3). Ahmad et al. [29] reported the solubility of sweet potato starches 7.8 ± 0.76%, almost corresponding to the solubility of the extracted *β*-glucan. It is of interest to point out that low solubility of *β*-glucan may reflect the strong binding between glucose molecules in *β*-glucan chain. Also, the low solubility of the *β*-glucan reflects that *β*-glucan hydrated very little. This could be one of the reasons for low swelling percentages. However, the solubility in water can be increased through modifying the *β*-glucan structure such as phosphorylation and sulfonation. In a separate experiment of this investigation, phosphorylation has dramatically improved the solubility of *β*-glucan (data not shown).

Table 4 summarized the least gelation concentration of *β*-glucan. The result shows that commercial *β*-glucan can form complete gel at 8.0% versus extracted *β*-glucan which was able to form gel at 4%. The difference might be due to the particle size and extraction process used in this experiment.

#### 3.2.5. Bile Acid Binding Activity

**Figure 7:**
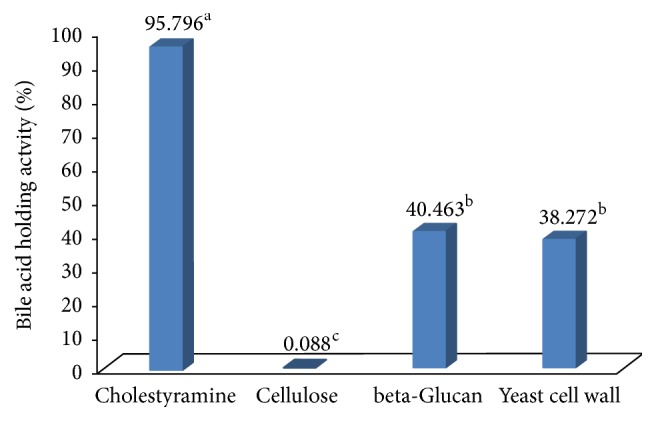
Showing the Bile acid holding activity. Cellulose was used as negative control; cholestyramine was used as positive control; all experiments were repeated three times (*n* = 3); extracted *β*-glucan and yeast cell wall exhibited similar bile acid binding activity (*P* ≥ 0.05). Different superscript letters, resulting from post hoc test after application of statistical model, show that treatments are statistically different from each other.

The results regarding the bile acid holding activity (in vitro) by the various components are shown in Figure 7. There was no significant difference (*P* ≥ 0.05) in the bile acid holding activity between yeast cell wall and *β*-glucan. The magnitude in improvement regarding bile acid binding by yeast *β*-glucan is better in this study compared to those reported for the barley and oat *β*-glucan (33%, 11.33%), respectively [30, 31]. The present results suggested that the fluctuation in bile acids binding levels is due to the differences in sources of *β*-glucan and that yeast *β*-glucan provides more protection than oat or barley *β*-glucan, due to their unique molecular structure.

#### 3.2.6. Antioxidant Property of *β*-Glucan

**Figure 8:**
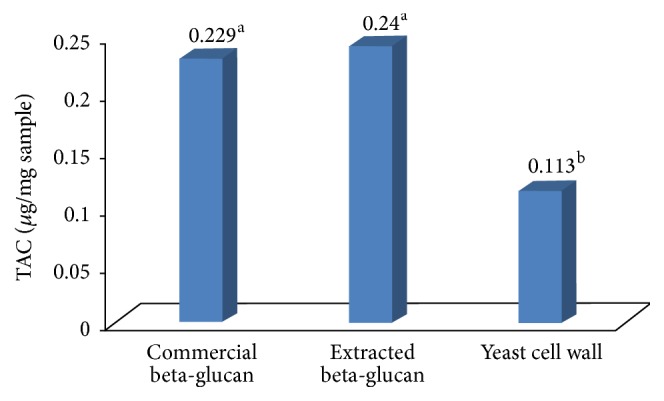
Showing total antioxidant capacity of commercial *β*-glucan; extracted *β*-glucan and yeast cell wall. All experiments were repeated three times (*n* = 3). Yeast cell wall showed comparatively lower (*P* ≥ 0.05) total antioxidant activity than commercial or extracted *β*-glucan. Different superscript letters, resulting from post hoc test after application of statistical model, show that treatments are statistically different from each other.

**Figure 9:**
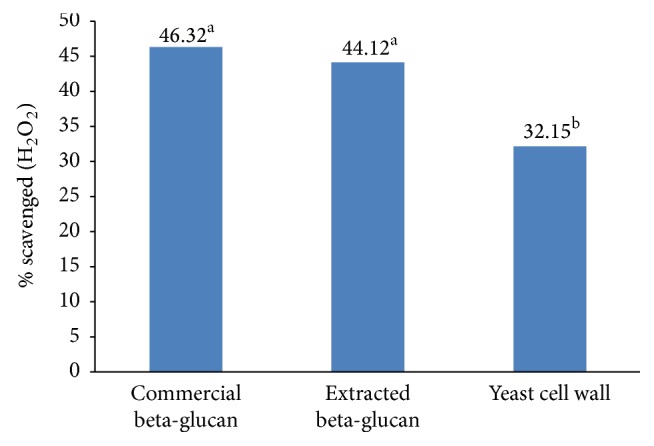
Showing scavenging activity (%) of commercial *β*-glucan; extracted *β*-glucan and yeast cell wall. Experiments were repeated three times (*n* = 3); scavenging activity (H_2_O_2_) of the yeast cell wall was comparatively lower (*P* ≤ 0.05) than commercial or extracted *β*-glucan. Different superscript letters, resulting from post hoc test after application of statistical model, show that treatments are statistically different from each other.

Total antioxidant capacity exhibited by the commercial and extracted *β*-glucan (Figure 8) was similar (*P* ≥ 0.05). The health promoting capability of *β*-glucan extracted from the yeast is comparatively higher than earlier report on baker yeast autolysate 0.080 ± 0.006 mg/mL [32]. Regarding the (-OH) neutralization, there was significant difference (*P* = 0.0147) in % scavenging activity between the groups. Commercial and isolated *β*-glucan have similar scavenging activity (*P* ≥ 0.05) against H_2_O_2_ clearance. However, yeast cell wall exhibited significantly lower H_2_O_2_ scavenging activity (Figure 9).

#### 3.2.7. In Vitro Glycaemic Response Evaluation Study

**Figure 10:**
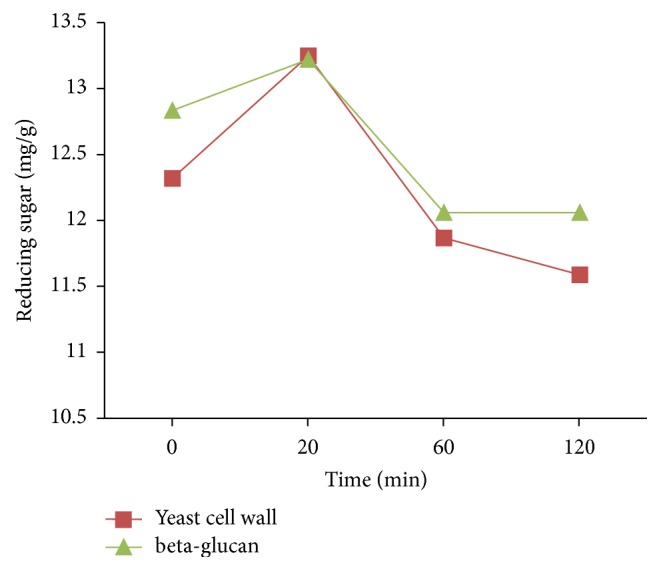
In vitro glycaemic response evaluations; describing the release of reducing sugar (mg/g) during in vitro digestion study. The experiment was repeated 3 times and the level of significance was *P* = 0.5581; *t*-test was used to compare the data using GraphPad Prism 5.

*β*-Glucan and yeast cell wall were analyzed for glycaemic response during in vitro digestion method. The experiments were repeated at least three times. The procedures of this experiment are based on the previously reported method [21] that mimics the human digestive track. The amount of sugar (mg) released to the environment from the samples (per gram) was varied between* yeast* cell wall and *β*-glucan samples but the effect was not significantly different (Figure 10). Both samples were found highly resistant to the digestive enzymes. The digestive enzymes were supposed to produce free sugar from the samples during 120 min digestion process. This clearly illustrated the benefit of slowly digestible polymers and that the extracted *β*-glucan possesses native structure that ensures resistance to digestion by stomach enzyme and thus due to this reason *β*-glucan could have released very limited sugar. This implies that *β*-glucan has low glycemic response and therefore might be used as useful nutraceutical agent in controlling metabolic chronic diseases [33].

### 3.3. Biological Characterization

#### 3.3.1. Effect of *β*-Glucan on Enzyme Myeloperoxidase Activity, Nitric Oxide, and Colon Weight

Dinitrochlorobenzene (DNCB) is a strong immune-genic, allergenic, and inflammatory agent, producing strong autoimmune response on contact with skin [34]. This response can be measured chemically and physically. The skin thickness is a type of physical estimation, caused by inflammation and immune cell infiltration. Chemically, the response can be measured through inflammatory enzyme, that is, myeloperoxidase activity. Our results have indicated significantly higher (*P* = 0.01) myeloperoxidase activity in the rats (group-2) versus DNCB treated rats supplemented with *β*-glucan diet (group 3 and group 4) and control rats, charged with normal diet. This finding implies that DNCB has produced strong effect though, but *β*-glucan diet was able to reverse the effects caused by DNCB. The effect of *β*-glucan @ 4% was better in terms of reducing myeloperoxidase activity and neutrophil count (%) in group 4 rats (4.465 ± 1.450) relative to group 3 rats (5.498 ± 2.208). The earlier report [35] on *β*-glucan from other sources has a similar action in DNCB treated rats, indicating *β*-glucan positive role in managing inflammation and autoimmune response.

Like myeloperoxidase, higher NO level may reveal inflammation and stress. Pulli et al. [36] reported an association of NO with the pathogenesis of inflammation, apoptosis, and tissue injury [37]. According to the results, significant differences (*P* ≤ 0.00) were found in NO levels across the treatment groups (Table 5). Normal rats in group 1 have presented statistically low NO values (9.125 ± 0.696) compared with group 2 rats which have significantly higher NO values (14.6 ± 1.052). However, this effect is significantly reversed in *β*-glucan treated groups (10.766 ± 0.462, 10.5 ± 0.834), respectively, in group 3 and group 4, suggesting the nutraceutical action of *β*-glucan. Although NO is a versatile substance, correcting vascular tone, still this molecule encourages toxic reactions against other tissues [38] and thus its elimination or reduction might be helpful in stressful condition.

Malondialdehyde (MDA) is considered to be a marker of lipid peroxidation [39]. The results of this study indicated significant difference (*P* ≤ 0.0001) in MDA level in different groups. Compared with control group (5.2 ± 0.501), group 2 rats exhibited significantly higher MDA content 10 ± 0.517 (Table 5). However, lipid peroxidation was markedly decreased in group 3 and group 4 rats (6.26 ± 0.546 mmol/L, 5.84 ± 0.349 mmol/L), respectively. Moreover, group 4 rats have the least MDA level compared with group 3 rats, and the effect was very close to group 1. These results suggest that 4% *β*-glucan supplementation is more effective against lipid peroxidation than 2% *β*-glucan supplementation. These findings are in collaboration with [40] that reported that dietary fibres are actively involved in improving lipid profile, reducing oxidative stress, and lowering systemic inflammation.

#### 3.3.2. Effect of *β*-Glucan on Total Cholesterol, Protein, and Blood Plasma Viscosity

There was significant difference found in total cholesterol (*P* ≤ 0.0002), proteins (*P* ≤ 0.0015), and blood plasma viscosity (*P* ≤ 0.0001) rats (Table 6). Group 3 rats exhibited the least cholesterol value 698 ± 35.440 mg/dL, though statistically similar to group 1 and group 4 rats. This shows that *β*-glucan supplementation can be used to reduce higher blood cholesterol. Our results are in collaboration with [39] that also reported the positive impact of dietary fibre. In fact, the least cholesterol content in group 3 rats showed that cholesterol might be oxidized or converted into MDA, as indicated by the higher level of MDA in group 3 rats. However, this observation needs further elucidation in long term dietary experimentation, under similar condition.

The protein level was drastically reduced in group 2 rats as compared to all other groups, indicating the high stress level in those rats. The protein content in group 1, group 3, and group 4 remained statistically similar (*P* ≥ 0.005) as shown in Table 6. Unlike lipids, proteins are stable and might require more time to be oxidized and thus the effect of DNCB on protein oxidation in group 3 and group 4 seems to be time and concentration dependent. Also, *β*-glucan supplementation in the latter groups might have provided immunity to the rats and therefore there was less oxidative damage to the proteins in group 3 and group 4 rats.

Regarding the viscosity, an essential characteristic of fluid relates to the medium/liquid composition and the interaction between the medium molecules. Blood plasma is composed of water, electrolytes, albumin, and fibrinogen and many more. Due to the strong interaction between these many different molecules, plasma represents high viscosity. It is well established that high blood viscosity is a serious threat for cardiovascular health. The group 2 rats exhibited significantly higher blood plasma viscosity (mPa·s), 3.44 ± 0.301 compared with control rats (1.814 ± 0.069) and *β*-glucan treated rats (group 3 and group 4). Also, there was a significant difference found between blood plasma viscosity of group 3 and group 4 (Table 6), as these two groups were treated with DNCB. Since group 2 was treated with DNCB, not given with *β*-glucan, it means the higher viscosity in this group, compared to others, was due to the sole effect of DNCB. Alternatively, enhanced free radicals may lead to increased blood viscosity.

Also, the higher levels of platelets (1082.45 ± 60.786%) in group 2 rats suggest a link between plasma viscosity and platelet level. There is positive correlation between plasma viscosity and fibrinogen, total serum protein, and total triglycerides (human study) [41]. Based on the study, our findings revealed that increasing concentrations of cholesterol, platelets, and neutrophils count in group 2 rats were the lead factor for enhanced viscosity.

#### 3.3.3. Effect of *β*-Glucan on Skin Thickness at Various Days

**Figure 11:**
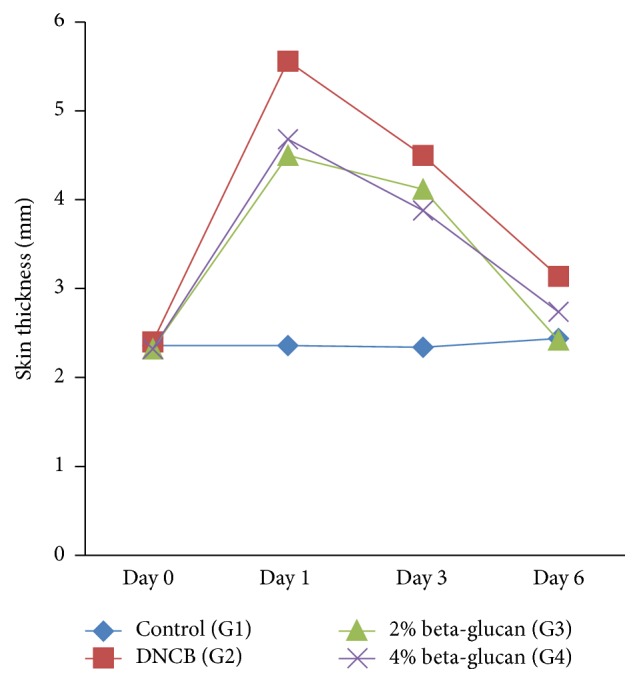
Effect of *β*-glucan on skin thickness (mm) in rats (*n* = 5). Skin response to DNCB exposure on various days; Day zero (0) = skin thickness (mm) similar in all groups; Day 1 = after exposure of DNCB on skin of all animals except control and the maximum thickness observed on Day 1; Day 3 and Day 6 = the skin thickness significantly reduced to control level.

The result regarding skin thickness demonstrated the effect of diet and time (*P* < 0.0001) on the skin thickness in different rat groups (Table 7, Figure 11). On Day zero (0) the skin thickness (mm) in all groups was found to be similar (*P* > 0.05) but then significantly increased (*P* = 0.0001) on Day 1 in group 2, group 3, and group 4, as DNCB has elicited strong inflammatory and immunogenic response. As can be seen, rats treated with the allergenic compounds together with *β*-glucan demonstrated strong retardation against inflammatory reaction in G3 and G4 rats versus group 2 rats. As far as the difference between group 3 and group 4 is concerned, it turns out that group 3 rats charged with 2% *β*-glucan have profoundly retarded skin thickness relative to group 4 rats.

The skin thickness (Day 3) significantly reduced in group 2, group 3, and group 4 compared to Day 1 while on Day 6, the skin thickness in all groups reversed to the skin thickness of control group (*P* > 0.05), though the skin thickness in group 2 and group 4 was found to be higher than the control group but not statistically different. Regarding the skin thickness, several lines of studies have shown [42] the clinical significance of polysaccharides rich PEE (*P. eryngii* extracts; this extract contains *β*-glucan) extract supplementation on the reduction in inflammatory response in the skin, due to the modulation in Th1 and The2 cell responses (lymphocytes) and prohibited skin lesions [43].

#### 3.3.4. Effect of *β*-Glucan on Blood Cells

There was a significant difference in white blood cells and platelets (*P* ≤ 0.0020), lymphocyte (*P* ≤ 0.0021), and blood neutrophil (*P* ≤ 0.0035) while red blood cells and hemoglobin concentration remained unchanged (*P* ≥ 0.1553, *P* ≥ 0.2941), respectively. According to the results, group 2 rats exhibited significantly higher (*P* < 0.05) WBCs versus others that reduced in group 3 and group 4 rats due to 2% and 4% *β*-glucan diets. The present findings are little higher than those previously reported by [2], 8.701 ± 122.67 WBCs per deciliter in control rats and 12.20 ± 147.21 per deciliter WBCs in rats fed with yeast supplemented diet (Table 8). Similarly, group 2 rats presented more platelets (10^3^/*μ*L), 1082.45 ± 60.786, compared with other groups. There was no significant difference in platelet levels between group 3 and group 4 rats, that is, 997.652 ± 33.315 and 973.228 ± 36.875, respectively. Moreover, the effect of 4% *β*-glucan (group 4) was found more on platelet reduction than 2% *β*-glucan (group-2) but the difference was not statistically significant (*P* ≥ 0.05).

Also, neutrophil count was markedly elicited in group 2 rats (21.694 ± 2.416%) compared with other groups, indicating the immunogenicity in this group. Regarding the dose, 4% *β*-glucan (group 4) exhibited remarkable reduction in neutrophil levels (18.204 ± 1.376%) versus group 3 (20.292 ± 1.524%) rats. These results demonstrated that the devastating effect of DNCB was partially neutralized in group 4 rats (Table 8).

Furthermore, group 2, group 3, and group 4 rats, respectively, presented nonsignificant reduction in hemoglobin (Hb) level versus group 1 rats. Furthermore, the decreased level in RBCs/Hb shows that DNCB at the current concentration was able to induce enough free radical production; due to this reason hemoglobin levels were degraded in the DNCB treated rats. The released free heme, according to [44], activates heme oxygenase-1 (HO-1, stress-response protein). This enzyme inhibits apoptosis, inflammation, and oxidative stress and thereby neutralizes prooxidant state.

The heme degradation pathway I is activated in response to HO-1 protein availability. Interestingly, HO-1 protein functionality is also based on enough free heme, inflammatory cytokines, and prostaglandins availability. This protein regulates blood iron level, thus protecting the tissue against oxidative stress. This observation explains the fact that *β*-glucan may activate HO-1 protein in the first place, followed by Hb degradation by the HO-1 protein, and that *β*-glucan may not directly activate heme degradation. The results obtained in the present study corroborate the results reported earlier [45] which speculated that HO-1 protein may induce genes that promote cytoprotection at the expense of Hb degradation. This may show the opportunity that *β*-glucan could be a safe food supplement and nutraceutical. Kazeem et al. [46] reported the effectiveness of nutraceutical interventions as anti-inflammatory agents.

The lymphocytes (%) were increased in group 2 rats (73.788 ± 1.906%) as compared with others. Kim et al. [47] reported that lymphocyte level increases in response to allergens, such as DNCB. Group 4 rats showed more anti-inflammatory effect, as reflected by low lymphocytes, 65.798 ± 3.260% (Table 8). In general, the effect of 4% *β*-glucan was found to be more pronounced in partial or complete retardation of inflammation in DNCB treated rats (group 4).

## 4. Conclusion

The maximum *β*-glucan (66%) from the yeast biomass can be effectively extracted using the optimized condition (1.0 M NaOH, 90°C, and pH 7.0). The isolated *β*-glucan is highly pure in this form compared with the commercial type as confirmed through FT-IR, SEM, DSC, and physicofunctional properties. The isolated *β*-glucan has promising bile acid binding (in vitro), antioxidant, and anti-inflammatory activity. Also, the in vivo experimentation regarding the cholesterol binding by *β*-glucan was encouraging. Furthermore, our results confirmed that yeast *β*-glucan is as effective as bacterial and plant based *β*-glucan against some of the inflammatory and oxidative stress markers studied in this investigation. In general, the effect of 4% *β*-glucan was found to be more pronounced in partial or complete retardation of DNCB produced effects. A similar, albeit far less noticeable, tendency was presented by the 2% *β*-glucan made diet. Overall, the efficiency of 4% *β*-glucan seems to be more satisfactory and therefore may be recommended in food formulation.

## Figures and Tables

**Table 1:** Formulation of diets (dry matter basis) per 100 grams. ^1,2^The American Institute of Nutrition Rodent Diets (AIN-93); *β*-glucan^3^ = *β*-glucan extracted from yeast in our laboratory.

Ingredients	Control normal diet	2% *β*-glucan diet	4% *β*-glucan diet
Corn oil	5.0	5.0	5.0
Mineral mixture^1^	5.0	5.0	5.0
Vitamin mixture^2^	1.0	1.0	1.0
Casein	10.0	10.0	10.0
*β*-glucan^3^	0.0	3.0	6.0
Corn starch	74.0	71	68
Pectin	5.0	5.0	5.0

**Table 2:** FT-IR analyses of yeast cells, commercial *β*-glucan, and extracted *β*-glucan; ^*∗*^according to [2]; ^*∗∗*^according to [10].

Band range (cm^−1^)	Yeast cell (cm^−1^)	Commercial *β*-glucan (cm^−1^)	Isolated/extracted *β*-glucan (cm^−1^)	Bands assignment
3000–3730^*∗*^	3409.873	3421.678	3440.964	OH groups^*∗*^
2820–3000^*∗*^	2924.460	3288.218; 924.265	3284.477; 2935.486	CH and CH_2_ stretching
1600–1700^*∗∗*^	1652.953	1652.914	1641.109; 1558.14	NH groups^*∗∗*^
970–1250^*∗*^	1077.852; 916.671	1076.391; 921.560	1079.780; 1045.339; 923.879	*β*-glycosidic, C-O group^*∗*^

**Table 3:** Composition (%; DW) and properties of commercial and extracted *β*-glucan. Mean values with *P* ≥ 0.05 are not significantly different.

Composition/property	Commercial	Extracted	*P* value
Carbohydrates	86.08 ± 0.9	88.74 ± 1.6	0.4753
Crude protein (CP)	4.8 ± 0.7	3.88 ± 0.2	0.1653
Ether extract (EE)	2.32 ± 0.0	3.90 ± 0.2	0.2545
Ash	1.24 ± 0.0	2.50 ± 0.2	0.1322
Water absorption capacity (mL/g)	6.066 ± 0.2	5.3 ± 0.360	0.50
Oil absorption capacity (mL/g)	1.76 ± 0.1	1.66 ± 0.1	1.00
Lose bulk density (g/mL)	2.17 ± 0.3	0.698 ± 0.0	0.014
Packed bulk density (g/mL)	1.42 ± 0.4	0.379 ± 0.0	0.003
Swelling percentage (%)	3.77 ± 0.2	4.833 ± 0.0	0.10
Water solubility (%)	8.6 ± 8.7	7.9 ± 6.0	0.65

**Table 4:** Least gelation concentration of commercial and extracted *β*-glucan from yeast.

Quantity (g/100 mL)	Commercial *β*-glucan	Extracted *β*-glucan
2	No gelation	No gelation
4	Partial gelation	Complete gelation
6	Partial gelation	Complete gelation
8	Complete gelation	Complete gelation
10	Complete gelation	Complete gelation
12	Complete gelation	Complete gelation
14	Complete gelation	Complete gelation
16	Complete gelation	Complete gelation
18	Complete gelation	Complete gelation
20	Complete gelation	Complete gelation

**Table 5:** Effect of *β*-glucan myeloperoxidase activity (skin), NO level, and colon weight in rats exposed to DNCB stress. Values are expressed as means ± SD; *n* = 5; mean values with similar superscript in a column are statistically nonsignificant (*P* ≥ 0.05); MPO activity = *µ*mol/min/mg DNA; NO level = *µ*mol/g; MDA = (mmol/L).

Groups	MPO activity (*P* = 0.001)	NO level (*P* = 0.00)	MDA	Colon weight (g) (*P* = 0.1114)
Group 1	3.613 **± **1.372^c^	9.125 ± 0.696^c^	5.2 ± 0.501^c^	2.487 ± 0.136^a^
Group 2	9.318 **± **1.567^a^	14.6 ± 1.052^a^	10 ± 0.517^a^	3.066 ± 1.074^a^
Group 3	5.498 **± **2.208^b^	10.766 ± 0.462^b^	6.26 ± 0.546^b^	2.430 ± 0.072^a^
Group 4	4.465 ± 1.450^cb^	10.5 ± 0.834^b^	5.84 ± 0.349^bc^	2.955 ± 0.827^a^

**Table 6:** Effect of *β*-glucan on total cholesterol, protein, and blood plasma viscosity of rats exposed to DNCB stress. Values are expressed as means ± SD; *n* = 5. Mean values with similar superscript in a column are statistically nonsignificant (*P* ≥ 0.05). Total cholesterol = mg/dL; protein = mg/mL; blood plasma viscosity = mPa·s.

Groups	Total cholesterol	Protein	Blood plasma viscosity
Group 1	709.2 ± 10.514^b^	7.34 ± 0.736^a^	1.814 ± 0.069^c^
Group 2	769 ± 23.537^a^	5.24 ± 0.553^b^	3.44 ± 0.301^a^
Group 3	698 ± 35.440^b^	6.52 ± 0.470^a^	2.408 ± 0.463^b^
Group 4	701 ± 19.849^b^	7.2 ± 0.352^a^	2.442 ± 0.394^b^

**Table 7:** Data regardingtheskin thickness in rats was measured through 2-factor ANOVA using Bonferroni posttests on different days (*n* = 5) at *α* = 0.005 using GraphPad Prism version 5. Control rats showed nonsignificant (*P* ≥ 0.05) variation in skin thickness throughout the study.

Day 0 versus Day 1					

Days	Day 0	Day 1	Difference	95% CI of diff.	*P* value

Control (G1)	2.3600	2.3600	2.3842	−0.94417 to 0.94417	*P* > 0.05
DNCB (G2)	2.4000	5.5600	3.1600	2.2158 to 4.1042	*P* < 0.001
2% *β*-glucan (G3)	2.3200	4.5000	2.1800	1.2358 to 3.1242	*P* < 0.001
4% *β*-glucan G4)	2.3200	4.6800	2.3600	1.4158 to 3.3042	*P* < 0.001

Day 0 versus Day 3					

Days	Day 0	Day 3	Difference	95% CI of diff.	*P* value

Control (G1)	2.3600	2.3400	−0.020000	−0.96417 to 0.92417	*P* > 0.05
DNCB (G2)	2.4000	4.5000	2.1000	1.1558 to 3.0442	*P* < 0.001
2% *β*-glucan (G3)	2.3200	4.1200	1.8000	0.85583 to 2.7442	*P* < 0.001
4% *β*-glucan (G4)	2.3200	3.8800	1.5600	0.61583 to 2.5042	*P* < 0.001

Day 0 versus Day 6					

Days	Day 0	Day 6	Difference	95% CI of diff.	*P* value

Control (G1)	2.3600	2.4400	0.080000	−0.86417 to 1.0242	*P* > 0.05
DNCB (G2)	2.4000	3.1400	0.74000	−0.20417 to 1.6842	*P* > 0.05
2% *β*-glucan (G3)	2.3200	2.4200	0.10000	−0.84417 to 1.0442	*P* > 0.05
4% *β*-glucan (G4)	2.3200	2.7400	0.42000	−0.52417 to 1.3642	*P* > 0.05

Day 1 versus Day 3					

Days	Day 1	Day 3	Difference	95% CI of diff.	*P* value

Control (G1)	2.3600	2.3400	−0.020000	−0.96417 to 0.92417	*P* > 0.05
DNCB (G2)	5.5600	4.5000	−1.0600	−2.0042 to −0.11583	*P* < 0.001
2% *β*-glucan (G3)	4.5000	4.1200	−0.38000	−1.3242 to 0.56417	*P* > 0.05
4% *β*-glucan (G4)	4.6800	3.8800	−0.80000	−1.7442 to 0.14417	*P* < 0.005

Day 1 versus Day 6					

Days	Day 1	Day 6	Difference	95% CI of diff.	*P* value

Control (G1)	2.3600	2.4400	0.080000	−0.86417 to 1.0242	*P* > 0.05
DNCB (G2)	5.5600	3.1400	−2.4200	−3.3642 to −1.4758	*P* < 0.001
2% *β*-glucan (G3)	4.5000	2.4200	−2.0800	−3.0242 to −1.1358	*P* < 0.001
4% *β*-glucan (G4)	4.6800	2.7400	−1.9400	−2.8842 to −0.99583	*P* < 0.001

Day 3 versus Day 6					

Days	Day 3	Day 6	Difference	95% CI of diff.	*P* value

Control (G1)	2.3400	2.4400	0.10000	−0.84417 to 1.0442	*P* > 0.05
DNCB (G2)	4.5000	3.1400	−1.3600	−2.3042 to −0.41583	*P* < 0.001
2% *β*-glucan (G3)	4.1200	2.4200	−1.7000	−2.6442 to −0.75583	*P* < 0.001
4% *β*-glucan (G4)	3.8800	2.7400	−1.1400	−2.0842 to −0.19583	*P* < 0.01

**Table 8:** Effect of *β*-glucan supplementation on blood parameters of rats exposed to DNCB stress. Values are expressed as means ± SD; *n* = 5. Mean values with similar superscript in a column are statistically nonsignificant (*P* ≥ 0.05); WBC = 10^3^/dL; platelet = 10^3^/*μ*L; neutrophil = %; RBCs = 10^6^/dL; Hb = g/dL; lymphocytes = %.

Groups	WBC	Platelets	Neutrophil	RBCs	Hb	Lymphocytes
G1	13.49 ± 0.647^b^	895.862 ± 39.477^c^	17.032 ± 0.3348^c^	9.84 ± 0.8406^a^	22.09 ± 14.635^a^	67.466 ± 3.259^bc^
G2	17.46 ± 1.335^a^	1082.45 ± 60.786^a^	21.694 ± 2.416^a^	8.406 ± 0.854^a^	14.002 ± 0.470^a^	73.788 ± 1.906^a^
G3	15.27 ± 1.139^b^	997.652 ± 33.315^b^	20.292 ± 1.524^ab^	8.644 ± 0.972^a^	13.154 ± 0.743^a^	70.778 ± 1.206^ab^
G4	15.124 ± 1.431^b^	973.228 ± 36.875^b^	18.204 ± 1.376^bc^	9.318 ± 1.012^a^	13.626 ± 0.780^a^	65.798 ± 3.260^c^
